# Roles of electrical impedance tomography in lung transplantation

**DOI:** 10.3389/fphys.2022.986422

**Published:** 2022-11-03

**Authors:** Hui Jiang, Yijiao Han, Xia Zheng, Qiang Fang

**Affiliations:** Department of Critical Care Medicine, The First Affiliated Hospital, Zhejiang University School of Medicine, Hangzhou, China

**Keywords:** electrical impedance tomography, lung transplantation, lung functional imaging, mechanical ventilation, lung perfusion

## Abstract

Lung transplantation is the preferred treatment method for patients with end-stage pulmonary disease. However, several factors hinder the progress of lung transplantation, including donor shortages, candidate selection, and various postoperative complications. Electrical impedance tomography (EIT) is a functional imaging tool that can be used to evaluate pulmonary ventilation and perfusion at the bedside. Among patients after lung transplantation, monitoring the graft’s pulmonary function is one of the most concerning issues. The feasible application of EIT in lung transplantation has been reported over the past few years, and this technique has gained increasing interest from multidisciplinary researchers. Nevertheless, physicians still lack knowledge concerning the potential applications of EIT in lung transplantation. We present an updated review of EIT in lung transplantation donors and recipients over the past few years, and discuss the potential use of ventilation- and perfusion-monitoring-based EIT in lung transplantation.

## 1 Introduction

Since Hardy et al. (1963) successfully performed the first human lung transplantation, lung transplantation has continued to develop for several decades. Lung transplantation has become the first-choice treatment for chronic, end-stage lung disease patients in whom medical therapy was ineffective (DeFreitas et al., 2021). For patients with severe functional impairments caused by respiratory diseases, lung transplantation can substantially improve quality of life and prolong survival (Arcasoy and Kotloff, 1999). There has been steady growth in the annual incidence of lung transplantations, with approximately 4,500 single and bilateral lung transplantations performed annually in adults since 2017 (Chambers et al., 2019). Based on records from the International Society for Heart and Lung Transplantation (ISHLT) International Thoracic Organ Transplant (TTX) Registry, a total of 67,493 adult recipient transplants were reported from January 1992 to June 2018 (Chambers et al., 2021). Despite progress in surgical techniques, immunosuppressive agents, and perioperative care, many early complications, such as primary graft dysfunction (PGD), threaten the pulmonary function and viability of the allograft. Delayed onset complications, such as chronic lung allograft dysfunction (CLAD), adversely impact the mortality and long-term outcomes of recipients (Meyer, 2018). As lung transplantation continues to develop, success will be determined by the ability to overcome obstacles, including donor shortage, appropriate candidate selection, PGD, and CLAD (Young and Dilling, 2019). They all concern lung function without exception, and assessing pulmonary function is an indispensable part (Tejwani et al., 2016).

**FIGURE 1:**
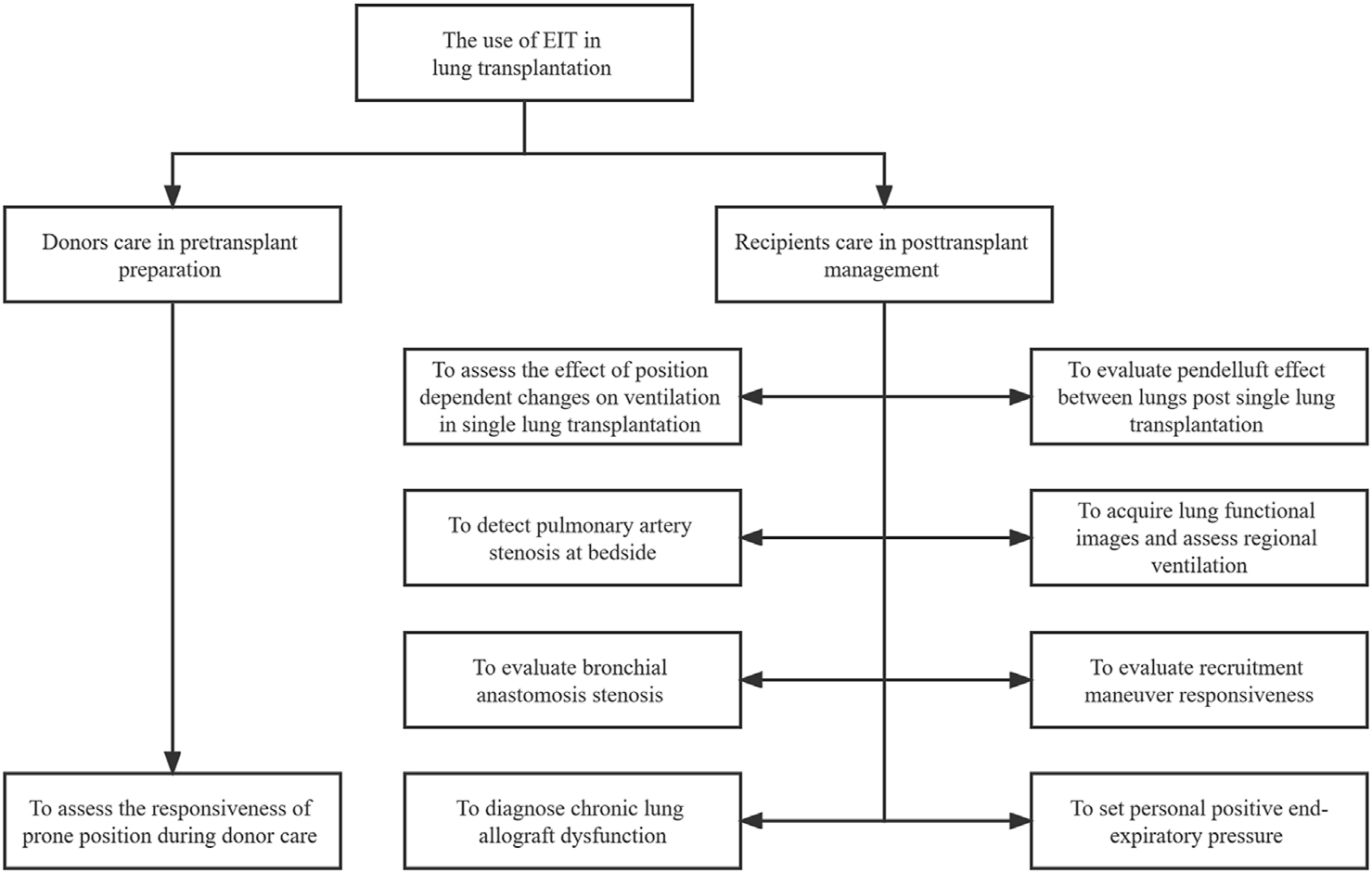
The illustrative figure on the use of EIT in lung transplantation. EIT: electrical impedance tomography.

After lung transplantation, patients usually require postoperative care and mechanical ventilation in an intensive care unit (ICU). Therefore, one of the most concerning issues for physicians is monitoring the graft’s pulmonary function (Fuehner et al., 2016). The conventional lung function imaging techniques may be inconvenient or impossible for critically ill patients in the ICU. Fortunately, a new technique has been developed. Electrical impedance tomography (EIT), a radiation-free, noninvasive, and real-time imaging tool, utilizes the changes in bioelectrical impedance to extract information and provides dynamic images of ventilation at the bedside (Frerichs et al., 2017). Since EIT allows frequent adjustment of ventilator settings, it is particularly useful in assessing heterogeneous gas distribution during mechanical ventilation (Shono and Kotani, 2019). The technique has proven to be useful in ventilator parameter optimization (Bachmann et al., 2018). Various studies have reported that EIT can be used for recruitment maneuvers (RM), positive end-expiratory pressure (PEEP) titration, lung volume estimation, and pulmonary perfusion (Zhao et al., 2010; Ambrósio et al., 2021; Xu et al., 2021). Since EIT was first available commercially in Europe in 2011, clinical research and case reports have been successively conducted all over the world (Shono and Kotani, 2019). However, few studies and cases have reported the practice of EIT in lung transplantation. In this review, we present an updated clinical review of EIT in lung transplantation donors and recipients over the past few years (Table 1) and discuss the potential use of ventilation and perfusion monitoring-based EIT in lung transplantation (Figure 1).

**TABLE 1:** Summary on research of EIT in lung transplantation.

Researchers	Research types	Subjects	Main findings of the research	Indications for EIT in lung transplantation	Limitations of the research
Caruana et al. (2010)	Case report	A patient treated with single lung transplantation for idiopathic pulmonary fibrosis	Pendelluft was detected using EIT between lungs after single lung transplantation during mechanical ventilation.	Pendelluft phenomenon detection	The finding lacked the confirmation of a randomized controlled trial
Jr et al. (2010)	Cohort study	Sixteen lung transplantation patients (6 bilateral, 5 unilateral for emphysema and 5 unilateral for fibrosis)	EIT can show dynamic lung images and be used to assess the regional ventilation in patients submitted to lung transplantation.	Regional ventilation assessment	The sample size of the study was small
Camargo et al. (2014)	Case report	A patient with bronchiolitis obliterans syndrome after bilateral lung transplantation	EIT seems to be helpful in differentiating bronchiolitis obliterans syndrome from a decrease in FEV_1_ caused by other reasons after lung transplantation.	Bronchial anastomosis stenosis evaluation	The finding lacked the confirmation of a randomized controlled trial
Guerin and Frerichs (2014)	Case report	A patient with lymphangioleiomyomatosis treated with single lung transplantation	A better picture of the correlation between lung function and structure can be obtained using EIT. EIT tends to provide images closer to the truth than X-rays and CT.	Functional lung imaging	The use was not confirmed
Ramanathan et al. (2016)	Case report	Six patients treated with single lung transplantation	Changes in regional ventilation may be associated with changes in position between transplanted and native lung.	Position-dependent changes in regional ventilation in single lung transplantation	The study involved patients with spontaneous breathing only, and the results cannot be translated to those under mechanical ventilation
			Ventilation of transplanted lung was observed to be better than that of native lung, especially in lateral positions		
Romero et al. (2016)	Case report	A single left lung transplantation recipient with emphysema-type COPD.	Recruitment maneuver was performed under EIT, and enabled the tidal volume distribution and the pressures required to ventilate the transplanted lung to be observed.	Recruitment maneuver responsiveness	The finding lacked the confirmation of a randomized controlled trial
Hart (2016)	Cohort study	Fifty lung transplantation patients	EIT is valid for the diagnosis of chronic lung allograft dysfunction in lung transplantation recipients.	Chronic lung allograft dysfunction diagnosis	No results of the study were disclosed
Sella et al. (2021a)	Cohort study	Six patients after bilateral lung transplantation (4 for pulmonary fibrosis, 1 for bronchiectasis and 1 for BOS)	EIT is a reliable tool for individualized PEEP setting in lung transplantation recipients, with similar to PEEP titration based on the best respiratory system static compliance.	PEEP titration	The finding lacked the confirmation of a randomized controlled trial
Son et al. (2021)	Case report	A lung donor after brain death	Prone positioning using EIT acutely improved the oxygenation of a brain-dead donor’s lungs with atelectasis. It may be a feasible strategy to improve the pulmonary function of a marginal lung donor.	Intervention during donor care before lung transplantation	The use was not confirmed
Zarantonello et al. (2021)	Case report	A single lung transplantation recipient with pulmonary artery stenosis	EIT has a potential role in identifying ventilation-perfusion mismatch caused by postoperative vascular complications and allows for noninvasive bedside detection and follow-up.	Bedside detection and follow-up of pulmonary artery stenosis	Locations of stenosis cannot be precisely positioned

EIT, electrical impedance tomography; FEV_1_, forced expiratory volume in one second; CT, computed tomography; COPD, chronic obstructive pulmonary disease; BOS, bronchiolitis obliterans syndrome; PEEP, positive end-expiratory pressure.

## 2 Basic principle and parameters of EIT

### 2.1 Basic principle

Different tissues possess distinct impedance characteristics, which are determined by specific compositions, such as fat, water, and electrolytes in the extracellular fluid (Lobo et al., 2018). Thoracic bioimpedance is significantly influenced by two cyclic procedures: ventilation and perfusion. Chest impedance varies on a large scale from residual capacity to total lung volume. However, perfusion causes a relatively small change from diastole to systole compared to ventilation (Bodenstein et al., 2009). To perform bioimpedance measurements, the electrode belt of EIT is tied around the thorax. The instrument determines the distribution of bioimpedance by injecting small alternating currents through surface electrodes and measuring potential differences between pairs of passive electrodes (Muders et al., 2010). According to Ohm’s law, the bioelectrical impedance between the injecting and measuring electrode pairs is obtained from the known applied current and the measured voltages. This process is repeated constantly around the entire thorax during a current cycle, and a series of results are used to reconstruct one cross-sectional EIT image (Maciejewski et al., 2021).

EIT can be used to assess lung perfusion distribution. The pulsatility method and the saline bolus injection method are the two methods of EIT used to assess lung perfusion. The pulsatility method is used to estimate pulmonary perfusion based on the measurement of pulsatile variation in pulmonary blood volume (Grant et al., 2011). Continuous information on pulmonary perfusion is retrieved by using cardiac and ventilation EIT signal separating techniques involving frequency-domain filtering, electrocardiogram (ECG) gating, and respiratory pause (Vonk Noordegraaf et al., 1998; Frerichs et al., 2009). The saline injection method reflects regional pulmonary perfusion by bolus injection of a high-conductivity contrast agent that causes changes in thoracic electrical impedance. After saline injection, impedance is significantly decreased in a certain lung region, thus suggesting that there is more contrast agent flow and indicating adequate perfusion. To reduce the interference of respiration with impedance, it must be implemented during respiratory pause. At present, the global thoracic impedance remains relatively unchanged, which better reflects the effect of saline angiography (Xu et al., 2021).

### 2.2 Basic parameters

The main view in EIT displays the following three parts: The pulmonary images, the impedance waveforms, and the digital panel. The pulmonary image is divided into dynamic image and end-expiratory image of the impedance variation. A dynamic image shows an animated EIT image that represents impedance changes against the baseline. The cross-section image can be set to four different regions of interest (ROI) according to the relative position in the thorax. Global impedance waveform shows the relative impedance changes over time, while regional impedance waveforms represent relative impedance changes of the defined ROI. At the right side of waveforms, regional tidal variations (TV) in percentage of the global TV occurring in each ROI are displayed as numeric values. The general parameters of EIT can be classified into the spatial and temporal distribution parameters. For the spatial distribution parameters, global tidal impedance variation (TV global) describes the global impedance change between beginning and end of inspiration in the breath. Likewise, regional tidal variation describes the regional impedance change within the corresponding ROI. End-expiratory lung impedance (EELI) infers the relative change in end-expiratory lung volume (Eronia et al., 2017). The center of ventilation (COV) describes the weighted geometric ventilation center, and a high level indicates that ventilation shifts to the dorsal side (Frerichs et al., 2020). The global inhomogeneity index (GI) reflects the extent of heterogeneous gas distribution in ventilation (Zhao et al., 2010). In the temporal distribution parameters, regional ventilation delay (RVD) and regional ventilation delay inhomogeneity (RVDI) contain regional pulmonary mechanics information derived from EIT. RVD associated with atelectasis areas shows a delayed time in the distribution of inspired air during the respiratory cycle (Muders et al., 2012). The delayed time required for the regional time-impedance curve to reach a certain threshold of its maximum impedance change. The RVDI is the standard deviation of the RVD in all pixels, which quantifies the time heterogeneity of the regional ventilation time course (Muders et al., 2010). Moreover, some EIT derived parameters indicate respiratory physiology and pathophysiology. For each breathing cycle, silent spaces are defined as pixels with impedance change of less than 10% of the maximum impedance change. Based on the relative position of the pixels to the COV, the silent spaces are divided into dependent silent space and non-dependent silent space (Spadaro et al., 2018). During ventilation, flow and airway pressure are recorded using a pneumotachograph, along with the EIT signal. Each regional pressure-volume (PV) curve corresponds to a row of pixels in the EIT image. The regional PV curve is fitted using the sigmoidal equation and the regional inflection points of each curve are mathematically determined (Scaramuzzo et al., 2019). Moreover, regional hysteresis was calculated as the difference between the expiratory and inspiratory limbs of the EIT-based regional PV curve (Scaramuzzo et al., 2020b).

## 3 The use of EIT in lung transplantation

### 3.1 Donor care in pretransplant preparation

In recent years, an overwhelming majority of transplanted lungs have come from brain-dead (BD) patients (Wong and Liu, 2021). If a BD donor has sustained injury due to inflammatory responses, catecholamine storms, and ischemia-reperfusion, then the lungs are usually unusable for transplantation (Avlonitis et al., 2003). There has been a lack of appropriate donor care, and a considerable number of potential donors do not meet ideal lung donor criteria. When lungs from insufficient-quality donors, also known as marginal donors, are transplanted and the overall mortality is adversely affected (Chaney et al., 2014). The ratio of the arterial partial pressure of oxygen to the fraction of inspired oxygen (PaO_2_/FiO_2_), as one of the main criteria for ideal lung donor, needs to be more than 300 mmHg (Van Raemdonck et al., 2009). However, atelectasis and hypoxemia are more likely to occur in BD donors due to the absence of spontaneous breathing and a cough response (Marklin et al., 2020). Therefore, active pulmonary management strategies, including RM, bronchoscopy, and lung-protective ventilation, could be used to reverse atelectasis and improve the utilization rate of donors (Marklin et al., 2021). Son et al. (2021) reported that use of the EIT-guided prone position converted an ineligible donor to a qualified donor. The PaO_2_/FiO_2_ of the patient was 342 mmHg when she was selected as a potential donor and later dropped to 49 mmHg due to severe atelectasis. The donor’s lung was unsuitable for transplantation. Within 4 h of being placed in the prone position, the atelectasis improved significantly. Tidal images on EIT showed that the status of ventral and dorsal ventilation returned to uniform, with matching bilateral ventilation. The donor’s lung was finally successfully transplanted due to gradual PaO_2_/FiO_2_ recovery. Another study similarly found that the prone position acutely improved the oxygenation of a hypoxemic BD donor’s lungs with atelectasis and resulted in more lungs being transplanted (Marklin et al., 2021). The prone position might be a feasible option for improving the condition of a marginal lung and enabling transplantation. Meanwhile, EIT can be used to observe the efficacy of the prone position in terms of improving of lung ventilation and perfusion (Zarantonello et al., 2020).

Improvement in PaO_2_/FiO_2_ has been considered a significant goal for restoring marginal lungs to the donor pool before transplantation. The common mechanisms leading to hypoxemia include ventilation/perfusion mismatch, hypoventilation and abnormal oxygen diffusion (Powner et al., 2005). The combined measurement of ventilation and perfusion by EIT could identify probable etiologies of acute respiratory failure at the bedside (He et al., 2021). EIT may be an available tool in etiology diagnosis and dynamic assessment in interventions during donor care before lung transplantation. Due to the prolonged supine position, atelectasis is common in cadaveric donors. Recruitment maneuvers are an important part of donor optimization, especially when oxygenation is lower than normal (Van Raemdonck et al., 2009). Yun et al. (2016) observed a discrepancy between regional lung reopening based EIT and oxygenation improvement after RM. EIT has the potential to evaluate the efficacy of RM by combining oxygenation measurements. These studies indicate that EIT can be used to assess the efficacy of donor maintenance dynamically to meet the ideal criteria before graft extraction. Moreover, EIT is a real-time evaluation imaging technique and does not require frequent patient transfer compared to X-ray and computed tomography. It has been suggested that EIT may be useful for ensuring the suitability of donor lungs; however, its use has not been confirmed.

### 3.2 Ventilation monitoring in posttransplant care

#### 3.2.1 Pulmonary functional image

Lobar torsion, bronchial anastomosis fistula, and various airway complications will most likely lead to inhomogeneous ventilation, hypoxia and/or dyspnea (Santacruz and Mehta, 2009; Lin et al., 2013). Whether in single or bilateral lung transplantation, it is essential to routinely monitor pulmonary ventilation during the postoperative period (Yeung and Keshavjee, 2014). EIT as a promising alternative for pulmonary functional imaging has recently attracted increasing attention. A case presented that EIT assessed the ventilation heterogeneity between the native and transplanted lung after single lung transplantation in a female recipient (Guerin and Frerichs, 2014). The recipient underwent single left lung transplantation in 2004 for lymphangioleiomyomatosis and developed CLAD with chronic respiratory failure requiring long-term home oxygen therapy. Ten years later, there was an acute deterioration in her condition. After progressing to acute respiratory distress, the patient underwent endotracheal intubation and ventilator connection with a low tidal volume strategy in the intensive care unit. The anteroposterior chest radiograph and the computed tomography scan showed a lack of ventilation in the left transplanted lung and overdistension in the right native lung. However, the waveforms of EIT showed the variation in thoracic impedance occurring almost exclusively on the left side of the chest, which meant there was no gas exchange in the right lung. Combining lung functional images of EIT (finding low ventilation in the native lung) and lung morphological images allowed the therapy team to identify the crux of the problem, namely treating the still ventilated transplanted lung. In this case, the main benefit of the EIT was the functional status information obtained regarding the native and transplanted lungs.

#### 3.2.2 Recruitment maneuver monitoring

Several complications of lung transportation, including PGD, acute rejection, and CLAD, adversely affect the prognosis of the recipient during the perioperative period (Whitson et al., 2007). PGD refers to a specific syndrome with acute lung injury comparable to acute respiratory distress syndrome (ARDS) within the first 72 h following transplantation (Snell et al., 2017; Swaminathan et al., 2021). In the case reported by Romero et al. (2016), a patient received left single lung transplantation for emphysema chronic obstructive pulmonary disease. To alleviate stage 3 PGD during the postoperative period, mechanical ventilation was performed using the Biphasic Positive Airway Pressure (BIPAP) model, which was set at a low tidal volume (290 ml) and a PEEP level of 8 cm H₂O to avoid lung overdistension. Subsequently, it was observed during ventilation monitoring using EIT that most of the gas was distributed in the native lung, and the ventilation of the left transplanted lung was insufficient. Recruitment maneuvers were performed under EIT to observe the tidal volume distribution and the alveolar opening pressure required to maintain ventilation of the transplanted lung. Although EIT cannot identify all disorders caused by insufficient ventilation, it can observe RM responsiveness directly and achieve a more homogeneous gas distribution.

#### 3.2.3 Regional ventilation inhomogeneity

The compliance of the transplanted lung is different from that of the native lung. The relative compliance depends on the damage to the transplanted lung and the underlying lung pathology characteristics (Anantham et al., 2005). Heterogeneity in ventilation after lung transplantation is common. Obviously, uneven ventilation distribution usually exists in single lung transplantation because the donor lungs from different individuals possess different respiratory mechanics (Lee et al., 2012). Likewise, bilateral lung transplantation may also lead to heterogeneous ventilation due to lung injury caused by PGD and acute rejection (Barnes et al., 2015). Jr et al. (2010) performed EIT in five single lung emphysema patients. Compared with the transplanted lung, different impedance variations and regional air trapping were observed in the native lungs. When adopting with conventional strategy, most of the tidal volume is transferred to the normal, more compliant lungs, which are disproportionately inflated (Anantham et al., 2005). This will lead to ventilator-induced lung injury and abnormal perfusion diversion. Hence, it is essential to set an appropriate PEEP to diminish ventilator-induced lung injury after lung transplantation. A decremental PEEP trial using EIT was performed in six bilateral lung transplantation patients (Sella et al., 2021a). The optimal PEEP was the most appropriate compromise between lung overdistension and collapse. After statistical analysis, the PEEP setting had a similar influence in EIT as in PEEP titration in achieving the best static compliance of the respiratory system.

It is well known that a pendelluft phenomenon refers to the transfer of gas from nondependent to dependent regions without changing the tidal volume. Nevertheless, it is assumed that pendelluft does not usually occur, except in two situations: violent spontaneous breathing efforts in ARDS and postinspiratory pause in heterogeneous lung units (Yoshida et al., 2013). Pendelluft leads to selective overdistension of the dependent lung regions and fails to efficiently contribute to gas exchange (Enokidani et al., 2021). Caruana et al. (2010) reported that a single lung transplant recipient with idiopathic pulmonary fibrosis was ventilated on synchronized intermittent mandatory ventilation with volume control (SIMV-VC). There was a significant difference between the filling time of the native and transplanted lung, determined as 1.07 s (*p* < 0.01, 95% confident internal: 0.90, 1.24) by EIT during SIMV-VC. The difference was greater than one second, which is considered clinically significant in filling times, indicating that the transplanted lung is filling while the native lung is emptying, known as the pendelluft phenomenon. EIT was used to determine the effectiveness of the ventilator mode and ventilation inhomogeneity in this study.

In addition, Ramanathan et al. (2016) assessed EELI and TIV in each lung in five different positions (the supine position, left lying position, right lying position, sitting, and standing) in six patients with single lung transplantation. The study showed that ventilation in the transplanted lung was significantly better than in the native lung, despite whether the transplanted lung was dependent in the lateral position. Firstly, it was a small sample study in which the pendelluft phenomenon in one patient’s lung strongly affected the results. When the outlier date from this subject was deleted, the difference in tidal variation became statistically significant for the most evaluated locations. Secondly, the above study only involved spontaneously breathing patients, and the results could not be translated into patients who underwent mechanical ventilation. Therefore, the findings of this study remain to be confirmed by the further studies.

#### 3.2.4 Complications detection

CLAD, a late complication in lung transplantation, is often characterized by a continuous decline in forced expiratory volume in one second (FEV_1_) for at least 3 weeks, limiting the 5-year survival to approximately 55% (Verleden et al., 2014). CLAD can be divided into two phenotypes: bronchiolitis obliterans syndrome (BOS) and restrictive graft syndrome (RAS) (Sato et al., 2013). Camargo et al. reported that the percentages of each lung ventilation in EIT were consistent with a bronchoscopy’s impression in bronchial anastomosis stenosis, suggesting that EIT might be useful in the differential diagnosis of BOS (Camargo et al., 2014). Moreover, Hart (2016) registered an intervention clinical trial to evaluate the diagnostic value of EIT in chronic rejections of lung transplantation. The study aimed to provide an accurate diagnostic method capable of distinguishing BOS from RAS compared to the current gold standard, FEV_1_, which can accurately stage BOS (Meyer et al., 2014). Furthermore, the study investigators considered providing EIT-based physiological data on lung transplant recipients with chronic rejection. The registered trial (Hart, 2016) was completed in 2018, but the corresponding published results have not been found.

### 3.3 Lung perfusion evaluation in posttransplant care

Ventilation/perfusion (V/Q) mismatch may occur after reperfusion in lung transplantation. The native lung is preferentially perfused because of the constricted vascular system of the transplanted lung, and it is preferentially ventilated due to its lower pulmonary compliance (Soluri-Martins et al., 2015). The assessment of lung perfusion is of valuable in posttransplant care. However, the variation in thoracic impedance from diastole to systole caused by perfusion is much smaller than that caused by ventilation (Bodenstein et al., 2009). EIT can be used to assess lung perfusion distribution by a distinct impedance comparison method. Zarantonello et al. (2021) reported EIT-based detection and follow-up of pulmonary artery stenosis in lung transplantation. A middle-aged male developed severe acute respiratory failure early after undergoing bilateral lung transplantation for idiopathic pulmonary fibrosis. EIT assessment showed a mismatch between ventilation and perfusion in the left graft due to reduced and delayed perfusion. The poor perfusion of the left lung led the researchers to focus on potential vessel stenosis. Severe left anastomotic pulmonary artery stenosis was confirmed by computed tomography pulmonary angiography. During the follow-up in the latter 5 days, EIT showed improvement in ventilation-perfusion matching with a resolution of the anastomotic stricture. According to the results of the literature search, this was the first report on the evaluation of lung perfusion in posttransplant lung care using EIT. Compared to the role of computed tomography pulmonary angiography in pulmonary artery stenosis, EIT allows repeated, nonradiative, and noninvasive bedside assessments. Therefore, EIT can potentially play a role in identifying ventilation-perfusion mismatch caused by postoperative vascular complications.

## 4 The future of EIT in lung transplantation

### 4.1 Personalized ventilation

The specific mechanical ventilation strategy for a lung transplant is still unclear, and there are no large-scale clinical randomized trials to confirm it. The mainstream view is to adopt a lung-protective strategy similar to ARDS patients (Barnes et al., 2015). It is believed that a higher level of PEEP is beneficial for ARDS patients (Amato et al., 2015). Nevertheless, it has been challenging to determine the optimal PEEP for mechanical ventilation of lung transplant recipients (Diamond and Ahya, 2014). A previous study showed that the low, medium and high tidal volumes had similar effects on short- and medium-term prognoses in lung transplantation recipients (Thakuria et al., 2016). Although a high PEEP level is associated with a high risk of pneumothorax and bronchial anastomotic complications, it is also associated with increased bronchial blood flow. Low PEEP may be associated with a higher driving pressure, and a high pressure will lead to poor physiological function, clinical prognosis, and pulmonary function (Thakuria et al., 2017). Therefore, a postoperative personalized PEEP setting is desirable to reduce ventilator-induced lung injury and minimize pulmonary complications in lung transplantation. Fortunately, EIT-guided PEEP titration at the bedside provides the possibility of individualized ventilation for recipients (Sella et al., 2021b). The first and most frequently used method monitors changes in regional lung compliance during a decremental PEEP trial. The optimal PEEP level is indicated by the best compromise between lung overdistension and collapse (Costa et al., 2009). The second method increases PEEP levels in maintaining EELI for assessing alveolar recruitment maneuvers (Eronia et al., 2017). The studies identified that EIT seems to be a promising bedside tool for personalized PEEP selection. The P-V curve has been suggested as a PEEP setting tool to explore changes in respiratory system compliance. However, it shows the global behavior of the lung without providing information on the regional lung mechanics. Additionally, it has been shown that regional inflection points derived from EIT reflect the heterogeneity of the lung and regional P-V curves obtained by EIT convey more sensitive information than global lung mechanics (Scaramuzzo et al., 2019). Compared with P-V curve PEEP titration, EIT-guided titration based on regional lung compliance is associated with improved driving pressure and survival rate in moderate to severe ARDS (Hsu et al., 2021). Based on the proportion of poorly or nonventilated lung units (silent spaces), the PEEP level suggested by EIT determines more ventilation homogeneity. It minimizes dorsal hypo-ventilated regions compared to transpulmonary pressure guided titration (Scaramuzzo et al., 2020a). The variation in EIT-derived dependent silent spaces induced by PEEP correlates well with lung recruitment measured by the P-V curve and could be used to set a personalized PEEP level. The deterioration of regional nondependent lung compliance suggests the potential role of EIT in identifying overdistention (Spadaro et al., 2018). Studies also described the optimal PEEP level as the value representing the lowest GI or the lowest RVD (Zhao et al., 2010; Muders et al., 2012).

Since asymmetrical lung disease leads to different airway resistance and lung compliance, the different ventilator strategies used in each lung are an innovative approach (Anantham et al., 2005). Once, researchers tried to apply different ventilation modes to each lung, designated differential lung ventilation (DLV) or one-lung ventilation (OLV). DLV is favorable to homogeneously distributed ventilation, V/Q mismatch decrease, and oxygenation improvement (Kremer et al., 2019). However, a feasibility study reported that EIT could play a role in immediately recognizing double-lumen tube misplacement in the contralateral main bronchus and the real-time assessment of DLV (Steinmann et al., 2008). Due to difficulties in catheter fixation, airway management, and sedation, DLV is burdensome to achieve in clinical practice.

For years, there has been the hypothesis that position affects lung region ventilation. In particular, the prone position has been considered an easy, inexpensive, and effective option for treating patients with ARDS (Pelosi et al., 2002). Moreover, the prone position to improve regional ventilation provides a novel alternative for reducing inhomogeneous ventilation after single lung transplantation (Marklin et al., 2020, 2021). A prospective study showed that EIT could dynamically assess the physiologic effects of the prone position using the tidal volume distribution and respiratory system compliance in dependent and nondependent regions, alveolar overdistension and collapse, and the increase in end-expiratory lung volume. With the above items, EIT might help identify patients who are more likely to obtain improved lung protection early by turning the prone position in ARDS (Dalla Corte et al., 2020). The study included severe ARDS patients and revealed that the prone position impacted global and regional ventilation using EIT monitoring during extracorporeal membrane oxygenation. This was reflected in the gradual redistribution of tidal volume and EELI from ventral to dorsal after the prone position (Franchineau et al., 2020).

### 4.2 Weaning

Prolonged mechanical ventilation is associated with an increased risk of in-hospital death in lung transplant recipients (Hadem et al., 2016). The ventilator weaning process should be attempted as soon as possible in recipients with hemodynamically stable and improved oxygenation (Kim et al., 2018). A spontaneous breathing trial (SBT) is usually performed to assess a patient’s eligibility for extubation. Arterial blood gas is used to determine the outcome of SBT in conventional SBT methods (Boles et al., 2007). Moreover, the possibility of weaning failure was low in patients with well-distributed ventral and dorsal ventilation, which was monitored by EIT-based regional intertidal gas distribution (Zhao et al., 2017). SBT failure was characterized in the early course of SBT by a more significant reduction in EELI and a higher level of GI in ventilation distribution than in patients who succeeded in SBT (Longhini et al., 2019). Bickenbach et al. (2017) recorded GI, TIV, EELI, and RVD at three points in time on SBT in patients with delayed weaning. It was shown that in patients with an initial GI > 41.5, an SBT was more likely to fail, with a sensitivity of 87.5% and a specificity of 60.9%, which suggested that EIT could predict the failure of weaning. Wang *et al.* found that the EIT may be a useful adjunctive tool for evaluating ventilator weaning using pre-SBT GI and SBT ROI_2_ (four ROIs were divided from the ventral to dorsal, with ROI_2_ being the second region) as predictors (Wang et al., 2021). In addition to weaning failure due to pulmonary system dysfunction, phrenic nerve injury can also lead to atelectasis, difficult weaning, and prolonged ventilation in lung transplantation (Hernández-Hernández et al., 2022). Moon et al. (2021) revealed that dynamic inhomogeneity of ventilation along the vertical axis of lungs using EIT could predict weaning failure regardless of diaphragm dysfunction. As a monitor to assess regional ventilation heterogeneity and its variations, EIT could be used to guide weaning and predict weaning failure in lung transplant recipients.

### 4.3 Perfusion monitoring

EIT can also be used to assess lung perfusion distribution, but the interference of cardiac perfusion needs to be ruled out. The pulsatility method is used to analyze the impedance variation in pulmonary vascular blood volume pulsatility to reflect pulmonary perfusion. However, pulsatility impedance does not directly reflect actual forward lung blood flow (Bluth et al., 2019). The accuracy of this method for assessing pulmonary perfusion has been questioned (Hellige and Hahn, 2011). It is susceptible to cardiac systolic and diastolic activities, airway pressure and distensibility of the small pulmonary vessels (Borges et al., 2012; Nguyen et al., 2012). Moreover, multiple animal experiments have indicated that saline bolus injection in EIT has a good correlation and consistency with single-photon emission computed tomography (SPECT) in pulmonary perfusion imaging (Fagerberg et al., 2009; Hentze et al., 2018). In recent years, growing attention has been given to the application of saline bolus EIT method in pulmonary perfusion imaging. Saline bolus injection for EIT perfusion imaging can be utilized for evaluating the V/Q matching condition in lung transplantation (He et al., 2020a). In recent years, growing attention has been given to the application of EIT in pulmonary perfusion imaging. Saline bolus injection for EIT perfusion imaging can evaluate the V/Q matching condition in lung transplantation (He et al., 2020a). Lung transplant recipients own an increased risk of pulmonary embolism (PE) compared to other hospitalized and postoperative patients (Krivokuca et al., 2011). He et al. showed that EIT-based ventilation and perfusion measures, including dead space, V/Q match, and intrapulmonary shunt percentage, could discriminate patients with acute PE from other patients with acute respiratory failure. The study suggested that contrast-enhanced EIT was potentially a promising bedside approach with good efficiency in PE diagnosis (He et al., 2020b). Idiopathic pulmonary arterial hypertension, a severe pulmonary vascular disease that does not respond to drug treatment, is one of the main indications for lung transplantation (Lordan and Corris, 2011). Meanwhile, rare pulmonary arterial hypertension (PAH) recurrence cases have been reported after lung transplantation (Soriano et al., 1999; Izbicki et al., 2005; Narula et al., 2014). Studies have shown that the impedance variation in lung perfusion is reduced in PAH in comparison with the normopressoric group. As it is closely related to hemodynamic characteristics, disease severity, and survival rate in patients with PAH, EIT might be a promising noninvasive technique for PAH diagnosis (Smit et al., 2006; Hovnanian et al., 2021). The potential validity of EIT in pulmonary perfusion assessment helps to identify and diagnose transplant complications from stenosis of pulmonary vascular anastomoses, such as PAH and PE.

The investigation of EIT in pulmonary perfusion imaging may reveal a new pathophysiological feature of ARDS. Pixels were classified as nonventilated if ventilation was less than 10% of the highest pixel-level value measured in the patient, and nonperfused was also defined as a similar method. The fraction of dead space and shunt corresponds to the percentage of nonventilated and nonperfused pixels in the total. The V/Q mismatch representation as to the sum of the dead space fraction and shunt fraction. Spinelli et al. (2021) found that V/Q mismatch was an independent predictor of death in ARDS patients with mechanical ventilation. Another study showed that a high dead space fraction could be a specific characteristic in patients with ARDS from coronavirus disease 2019 (Mauri et al., 2020). In recent years, Pavlovsky et al. (2022) developed a new EIT-based algorithm that resembles the multiple inert gas elimination technique (MIGET). The algorithm can be used to quantify V/Q mismatch and PEEP response, which may be a sensitive marker of ARDS severity.

### 4.4 Others aspects

Pneumothorax can occur spontaneously after lung transplantation and/or arise in patients receiving long-term mechanical ventilation (Slebos et al., 2001; Yuzuak et al., 2021). The use of EIT can be used to diagnose pneumothorax that occurs during recruitment maneuvers. Pneumothorax was manifested by a sudden increase in impedance in the affected region and a disproportionate amount of ventilation to the increase in PEEP. Since the technique only shows a variation in impedance, the affected area was no longer ventilated after pneumothorax, and the ventilation image was lost (Morais et al., 2017). Pleural effusions are prevalent phenomena after transplantation (Joean et al., 2021). Moreover, the validity in evaluating pleural effusion and emptying was also presented in EIT (Omer et al., 2021). The regional out-of-phase impedance changes are associated with the occurrence of pleural effusions (Becher et al., 2018). The out-of-phase impedance changes were characterized by a decrease in the impedance of certain areas during inspiration, followed by an increase during exhalation, which was contrary to normal conditions. After pleural effusion evacuating, an increase in end-expiratory impedance can be observed using EIT (Rara et al., 2020).

## 5 Limitations

For decades, imaging techniques have been used to detect abnormalities, monitor function, and diagnose diseases. Advances and the wide availability in lung imaging techniques have provided new insights into pulmonary structure and function and their alterations. More recently, functional lung imaging has been performed using positron emission tomography (PET), SPECT, and hyperpolarized gas magnetic resonance imaging (MRI) (Mirsadraee and van Beek, 2015; Bajc et al., 2019). PET and SPECT have higher image resolution than EIT, their application in lung transplantation is limited by radiation. The current protocols of hyperpolarized gas MRI rely on a fixed breath-holding movement, which depends on the patient’s cooperation (Fain, 2020). Whether radionuclides or hyperpolarized inert gases, these have been regarded as costly and hard-to-obtain materials. Moreover, it is unrealistic that transferring the patients, who received a lung transplant and connecting a ventilator, to the CT or MRI rooms. Because of its small size, portability, no need for radiation, and dynamic imaging ability, the EIT device is especially suitable for ventilation monitoring at the bedside in patients who are mechanically ventilated after lung transplantation. However, the limitations and constraints of this technique need to be expressly noted. First, the spatial resolution in EIT remains low because each pixel contains information about many alveolar unit impedances. An electric current is applied to the body through the electrodes, and then the voltage generated between the other electrodes is measured and recorded. Since the current is not limited to a horizontal plane, EIT obtains an image of an impedance change of approximately 5 cm cross-section of the thorax. One of the limitations of this principle is the low resolution. To improve this resolution, the number of bound electrodes could be increased (Tang et al., 2002). However, the standard available electrode belt on the market is 16 or 32 electrodes, and circumventing this issue requires more breakthroughs in design. Second, modifying the electrode belt position could lead to inconsistencies between the considered lung regions in multiple measurements. To reliably assess the ventilation and perfusion distribution, the belt should always be bound in a fixed position on the thorax (Sella et al., 2021b). The electrode belt should not be attached to damaged and inflamed skin. It is noted that the incision of the lung transplant coincides with the suggested binding region of the EIT electrode band, especially for double lung transplantation. The fractured state of the surgical incision affects the extraction of the electrical signal from the electrode band. Long periods of bondage or the application of conductive gels may lead to incision infection with delayed healing. Strict disinfection of the electrode belt at each operation may be beneficial for this problem. It is common for lung transplant recipients to have a drainage tube placed in the chest in the postoperative period (Joean et al., 2021). Excessive pleural effusion and severe pneumothorax may limit ventilation and affect the accuracy of ventilation assessment in EIT imaging of lung transplantation (Becher et al., 2018; Rara et al., 2020). However, as the chest tube drainage amount gradually decreases, this adverse impact may be alleviated with the appropriate timing of EIT operations. More reliable studies are needed to confirm EIT’s ability to distinguish between atelectasis, pleural effusion, and pneumothorax after lung transplantation (Zhou et al., 2022). Furthermore, other situations deserve attention to avoid adverse effects. The image quality obtained by EIT is poor in morbidly obese patients with a body mass index greater than 50. In addition, EIT is sensitive to body movement, and it may be unreliable to apply it to lung recipients with chaotic body movements. If lung transplant recipients is equipped with a pacemaker or need to defibrillate in the rescue, the use of EIT should be carefully considered, and its validity will be compromised (Lobo et al., 2018).

## 6 Conclusion

In summary, the application of EIT in lung transplantation, including lung donor care, pulmonary functional imaging, recruitment maneuvers and ventilation heterogeneity monitoring, postoperative complication detection and lung perfusion evaluation, has gradually increased. EIT may be a valuable technique that will be especially useful in lung transplantation, including but not limited to personalized ventilation, weaning, and perfusion monitoring. Indeed, the practice of EIT is still in its infancy, and the further studies are needed to further determine the potential use of EIT in lung transplantation.
